# Title: Understanding a Low Vitamin D State in the Context of COVID-19

**DOI:** 10.3389/fphar.2022.835480

**Published:** 2022-03-04

**Authors:** James Bernard Walsh, Daniel M. McCartney, Éamon Laird, Kevin McCarroll, Declan G. Byrne, Martin Healy, Paula M. O’Shea, Rose Anne Kenny, John L. Faul

**Affiliations:** ^1^ Mercer’s Institute for Successful Ageing, St James’s Hospital, Dublin, Ireland; ^2^ Department of Medical Gerontology, School of Medicine, Trinity College Dublin, Dublin, Ireland; ^3^ School of Biological and Health Sciences, College of Sciences & Health, Technological University Dublin, Dublin, Ireland; ^4^ Medicine Directorate, St. James’s Hospital, Dublin, Ireland; ^5^ Department of Clinical Biochemistry, St James’s Hospital, Dublin, Ireland; ^6^ Department of Clinical Biochemistry, Galway University Hospitals, Galway, Ireland; ^7^ School of Medicine, National University of Ireland Galway, Galway, Ireland; ^8^ James Connolly Memorial Asthma Research Centre, Royal College of Surgeons in Ireland, Connolly Hospital Blanchardstown, Dublin, Ireland

**Keywords:** SARS-CoV-2 infection, vitamin D, vitamin D supplementation, immunity, Bradford-Hill criteria, disease severity, causation, older adults

## Abstract

While a low vitamin D state has been associated with an increased risk of infection by SARS-CoV-2 in addition to an increased severity of COVID-19 disease, a causal role is not yet established. Here, we review the evidence relating to i) vitamin D and its role in SARS-CoV-2 infection and COVID-19 disease ii) the vitamin D status in the Irish adult population iii) the use of supplemental vitamin D to treat a deficient status and iv) the application of the Bradford-Hill causation criteria. We conclude that reverse causality probably makes a minimal contribution to the presence of low vitamin D states in the setting of COVID-19. Applying the Bradford-Hill criteria, however, the collective literature supports a causal association between low vitamin D status, SARS-CoV-2 infection, and severe COVID-19 (respiratory failure, requirement for ventilation and mortality). A biologically plausible rationale exists for these findings, given vitamin D’s role in immune regulation. The thresholds which define low, deficient, and replete vitamin D states vary according to the disease studied, underscoring the complexities for determining the goals for supplementation. All are currently unknown in the setting of COVID-19. The design of vitamin D randomised controlled trials is notoriously problematic and these trials commonly fail for a number of behavioural and methodological reasons. In Ireland, as in most other countries, low vitamin D status is common in older adults, adults in institutions, and with obesity, dark skin, low UVB exposure, diabetes and low socio-economic status. Physiological vitamin D levels for optimal immune function are considerably higher than those that can be achieved from food and sunlight exposure alone in Ireland. A window exists in which a significant number of adults could benefit from vitamin D supplementation, not least because of recent data demonstrating an association between vitamin D status and COVID-19. During the COVID pandemic, we believe that supplementation with 20-25ug (800–1000 IU)/day or more may be required for adults with apparently normal immune systems to improve immunity against SARS-CoV-2. We expect that higher monitored doses of 37.5–50 ug (1,500–2,000)/day may be needed for vulnerable groups (e.g., those with obesity, darker skin, diabetes mellitus and older adults). Such doses are within the safe daily intakes cited by international advisory agencies.

## Introduction

### Vitamin D Metabolism and Measurement

**FIGURE 1:**
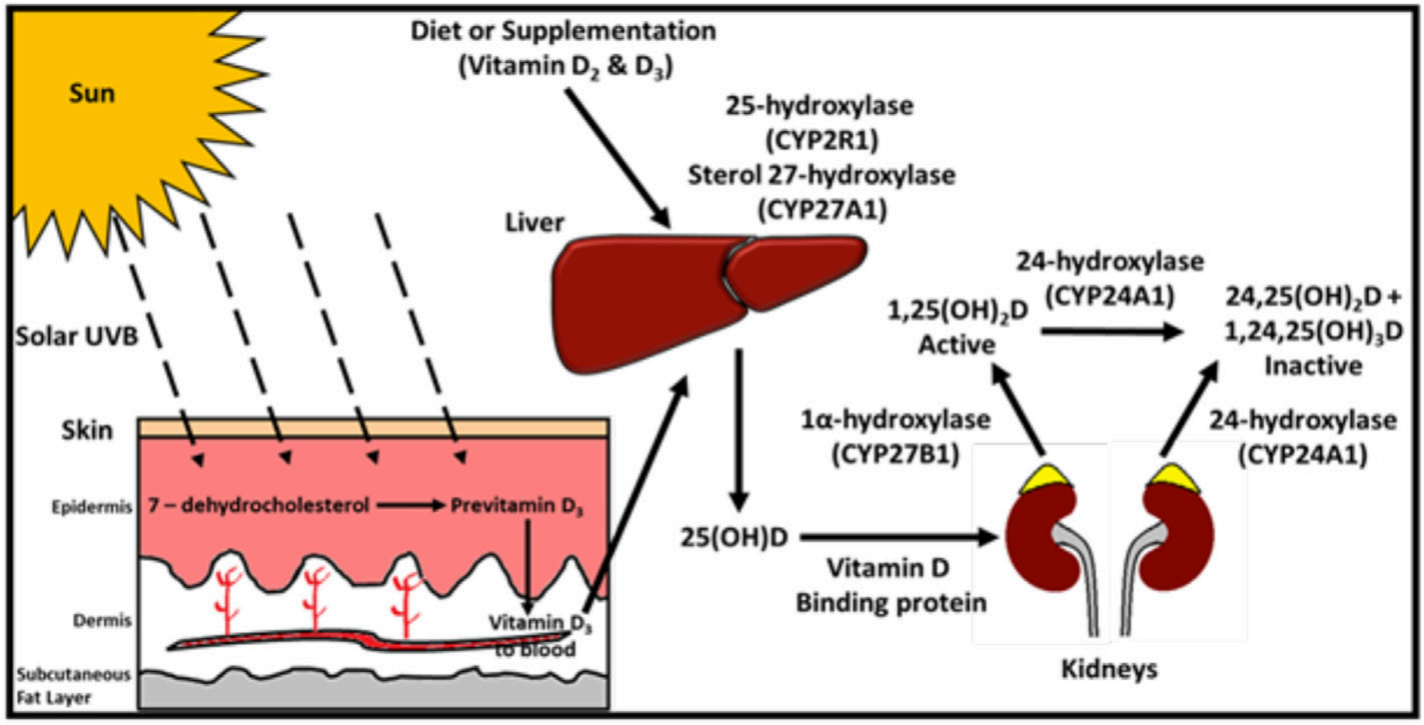
Vitamin D Metabolism: UVB radiation penetrates the skin, converting 7-dehydrocholesterol to pre-vitamin D_3,_ which is rapidly converted to vitamin D_3_. Vitamin D_3_ is transported through the circulation to the liver. Dietary vitamin D_2_ and D_3_ are transported from the intestine to the liver by chylomicrons (plasma and lymph). In the liver, vitamin D is hydroxylated to 25(OH)D, mediated by CYP2R1 (cytochrome P450 [CYP] enzyme). Once released into the circulation 25(OH)D binds to vitamin D binding protein and is transported to the kidneys and other tissues. In the proximal tubules of the kidney, 1α-hydroxylation (CYP27B1) of 25(OH)D results in the production of the active vitamin calcitriol (1.25(OH)_2_D). 1.25(OH)_2_D induces the expression of the enzyme 24-hydroxylase encoded by the CYP24A1 gene which catalyses the conversion of 25(OH)D and 1.25(OH)_2_D to the inactive 24-hydroxylated products, 24.25(OH)_2_D and 1,24,25(OH)_3_D respectively. Reproduced with permission from: Griffin TP, Bell M, Robinson S et al. (2017) Vitamin D and vitamin D deficiency in Ireland - a call to action. UPDATE Endocrinology and Diabetology [Internet]. 2017[37–40 pp.]. Available from: https://www.medicalindependent.ie/100980/update_ endocrinology__diabetology. Accessed 10.01.22).

The physiological metabolism of vitamin D derived from oral sources (diet and supplements) and cutaneous synthesis and the sequential hydroxylation steps converting vitamin D to 25(OH)D and through to the final production of the active hormone 1.25(OH)_2_D (Bikle, 2014) is schematically presented in Figure 1. Given its high circulating concentration (10^3^ times that of the active hormone) and long half-life, 25(OH)D is the internationally recognised marker for assessing vitamin D status. It reflects both dietary intake and cutaneous synthesis (Holick and Chen, 2008) and is the measurement of choice for clinicians (Holick, 2017). All major epidemiological studies related to vitamin D are based on its measurement. In addition, assays for 25(OH)D are standardised and traceable to international reference methods (Tai et al., 2010).

Although the bulk of research on vitamin D physiology involves analysis of 25(OH)D and 1.25(OH)D, recent work has demonstrated other metabolic pathways that may be important for vitamin D function. These include pathways incorporating the hydroxylation enzyme CYP11A1. This enzyme catalyses the first step in the steroidogenic pathway, the conversion of cholesterol to pregnenolone. It is now recognized that substrates other than cholesterol, including vitamin D, can be hydroxylated by CYP11A1 in positions other than 25- and 1α- (Slominski et al., 2012, 2015). The presence of CYP11A1 has been detected in both adrenal and extra-adrenal tissues including the skin, placenta, gonads, intestine and cells of the immune system (Slominski et al., 2021). Several products of CYP11A1 activated vitamin D hydroxylation have been identified including 20-hydroxyvitamin D (20(OH)D) which exhibits biological activity and has non-calcaemic, anti-proliferative, anti-inflammatory and anti-cancer properties. 25(OH)D, on the other hand, does not exhibit biological activity and must be 1α-hydroxylated by CYP27B1 (expressed in most cell types) to become bioactive. CYP11A1 is also expressed in many cells and, depending on the cell lineage, can initiate extra-adrenal corticosteroid biosynthesis or production of novel vitamin D hydroxy metabolites (Slominski et al., 2020a) including 20(OH)D, the most bioactive of these derivatives. With its non-calcaemic and anti-inflammatory properties 20(OH)D may have potential as an anti-COVID-19 therapeutic agent (Slominski et al., 2020b). As circulating vitamin D from dietary, supplemental or cutaneous UV synthesis is preferentially hydroxylated at the 25-hydroxy position in the liver, however, this requires the examination of alternative routes for exogenous 20(OH)D administration. Further discussion of these vitamin D hydroxyl derivatives can be found in two recent reviews (Jenkinson 2019 and; Tuckey et al., 2019). The overall contribution of these hydroxylated products to vitamin D physiology and metabolic functions continues to be investigated.

### Does COVID-19 Lower Serum Vitamin D Levels?

Vitamin D deficiency is prevalent worldwide (Lips et al., 2021) and low vitamin D status is associated with severe COVID-19 disease as described in over 900 papers from a variety of institutions and clinical settings (Ali, 2020; Bilezikian et al., 2020; Faul et al., 2020; Grant et al., 2020; McCartney and Byrne, 2020; McCartney et al., 2020). The observed associations support the hypothesis that a low vitamin D state impairs the human immune system, leading to poor clinical outcomes. A counter-argument exists however, whereby the observed associations might be explained by: (i) reverse causality, whereby severe COVID-19 lowers serum 25(OH)D levels (ii) the confounding effects of race, chronic kidney disease, advanced age, and obesity, all of which have long been associated with a low vitamin D state and have now been identified as significant risk factors for severe COVID-19 disease (Meltzer et al., 2021) and (iii) reliance on observational data in the absence of well-designed randomised controlled trials to confirm a therapeutic benefit of giving vitamin D to patients with established COVID-19.

A reduction in serum vitamin D might occur during the inflammatory process of severe COVID-19 disease. Indeed, researchers have studied whether systemic inflammation lowers circulating 25(OH)D levels (using the experimental human endotoxemia model, a standardised, controlled and reproducible model of systemic inflammation in humans). (Smolders et al., 2020). The observed acute reduction in 25(OH)D (from an average of 30 nmol/L to 23.6 nmol/L over 3 hours) is probably not large enough to explain the significantly lower serum 25(OH) D levels that are described in many patients with severe COVID-19 (Faul et al., 2020; Jain et al., 2020), but longer periods of inflammation might produce greater reductions. Average serum 25(OH) D levels less than 30 nmol/L are observed in patients with severe COVID 19 and ARDS while patients with COVID-19-related pneumonia (*i.e.,* not requiring mechanical ventilation) have 25(OH)D levels of less than 50 nmol/L. In Wuhan, China, a cross-sectional study of 335 COVID-19 patients and 560 controls with 25(OH)D measured before the SARS-CoV2 pandemic revealed that patients with COVID-19 had a higher prevalence of vitamin D deficiency (<30 nmol/L) on admission than that observed in the 2018–2019 control groups (65.1 vs. 40.7%; *p < 0.0001*) even after adjusting for sex, BMI and presence of comorbidities (*p* = 0.014). Additionally, serum vitamin D levels were found to be inversely correlated with disease severity; 82% of patients with severe COVID-19 presented with vitamin D deficiency compared to 60% of non-severe COVID-19 patients (*p* = 0.0004). (Luo et al., 2021). Moreover, even when measures of serum 25(OH)D are unavailable, it appears that a low vitamin D state precedes the onset of severe disease rather than the reverse (Annweiler et al., 2020). For example, in a study of nursing home residents who received a large bolus of vitamin D3 (80,000 IU) less than 1 month before becoming infected, when compared to those who received a bolus more than 1 month prior, the full-adjusted hazard ratio for mortality according to vitamin D3 supplementation was HR = 0.11 [95% CI: 0.03; 0.48], *p = 0.003*, supporting the hypothesis that low vitamin D status precedes severe disease, rather than the reverse (Annweiler et al., 2020). This theory that low vitamin D levels precede more severe COVID-19 outcomes is also strongly supported by a recent systematic review and meta-analysis incorporating eight studies in which serum 25(OH)D was measured either within 1 day of admission (4 studies) or up to 3–12 months prior to admission (4 studies). After correction for age, sex and presence of diabetes mellitus, this study reported a significant inverse correlation between serum 25(OH)D measured at or prior to admission and in-hospital COVID-19 mortality (Borsche et al., 2021). Studies that compare serum vitamin D levels immediately prior to infection with serum vitamin D levels soon after infection with SARS-CoV-2 should provide valuable information on the acute effect, if any, of SARS-CoV 2 infection, or COVID on serum vitamin D levels. Of course, the association of severe COVID-19 with several conditions that pre-dispose to a low vitamin D state also supports the theory that low vitamin D status causes more severe COVID-19, rather than the reverse. When ICU patients with a low vitamin D state were treated with vitamin D3 (a 200,000 IU bolus, followed by 10,000 IU per day for 10 days), the percentage of patients with serum 25(OH)D levels greater than 50 nmol/L increased from 34% to at least 58%, suggesting that serum 25(OH) D levels are dependent on vitamin D intake even in critically ill patients (Notz et al., 2021). Thus, reverse causality probably makes only a small contribution to low vitamin D states in the setting of COVID-19.

### What is the Association Between Vitamin D Status and SARS-CoV-2 Positivity?

Meltzer et al., investigated a large cohort of patients (n = 4,314) that were tested for COVID-19 of whom 499 had 25(OH)D levels measured in the year before testing, and determined that COVID-19 positivity was associated with increasing age (RR (age<50.1.05,*p* < 0.021; RR (age≥50) = 1.02,*p* < 0.064), non-white race (RR = 2.54,*p* < 0.01) and being likely vitamin D deficient (RR = 1.77,*p* = 0.02) compared to likely vitamin D sufficient. Predicted COVID-19 rates in the vitamin D deficient group were 21.6% (95% CI, 14.0–29.2%) vs 12.2% (95% CI, 8.9–15.4%) in the sufficient group (Meltzer et al., 2020a). A further cohort study by these authors comprising 489 patients who had 25(OH)D levels measured in the year prior to COVID-19 testing, reported the relative risk of testing positive for SARS-CoV-2 to be 1.77-fold greater and statistically significant for patients with low vitamin D status (<50 nmol/L) compared to those with adequate vitamin D levels (Meltzer et al., 2020b). In the US, a large retrospective observational study during the period of March to June 2020 of over 190,000 patients with diverse ethnic backgrounds revealed mean infection rates were significantly higher in subjects with low vitamin D state (<50 nmol/L) (mean infection rate = 12.5%; 95% CI: 12.2–12.8%) and in those with intermediate 25(OH)D3 levels: (75–85 nmol/L) (mean infection rate = 8.1%; 95% CI: 7.8–8.4%) than in those with higher 25(OH)D levels (>137 nmol L ^−1^) (mean infection rate = 5.9%; 95% C.I. 5.5–6.4%) (*p < 0.001*) (Kaufman et al., 2020). However, not all studies show this magnitude of association. For example, in a recent large study of almost 15,000 subjects from Brazil (2,345 infected with SARS CoV2 and 11,585 not infected), levels of serum 25(OH)D did not differ between the groups (*p* = 0.08) [mean 25(OH)D of 72 ± 54 nmol/L in infected subjects compared to 74 ± 45 nmol/L in the non -infected]. Conversely, in Sicily, when 50 patients infected with SARS CoV-2 were compared to 100 non-infected control patients (matched for age, gender, and BMI), median serum levels of 25(OH)D were significantly lower (31 vs. 51.5 nmol/L; *p* < *0.001*) in patients than in controls (Gaudio et al., 2021), a difference very similar to that observed between 27 Swiss SARS-CoV-2 positive patients and their 80 SARS-CoV-2 negative counterparts early in the pandemic (D’avolio et al., 2020). In England, in a study of 104 patients, socially deprived patients with vitamin D levels ≤34.4 nmol/L were most likely to be infected with SARS-CoV-2 and even more so if aged >72 years (OR: 19.07, 95%CI: 1.71–212.25; *p = 0.016*) (Livingston et al., 2021). In Saudi Arabia, serum 25(OH)D levels were significantly lower among 150 infected patients compared to 72 non-infected patients even after adjusting for age, sex and body mass index (BMI) (35.8 ± 1.5 nmol/L vs. 42.5 ± 3.0 nmol/L; *p* = *0.037*) (Alguwaihes et al., 2021). Meltzer et al. (2021), in a study of 4,638 participants, found that the risk of having Sars-CoV-2 positive results in Black individuals was 2.64-fold greater with 25(OH)D concentrations of 30 < 40 ng/ml. However, there were no significant associations found between vitamin D levels and COVID-19 positivity rates in White individuals. A recent meta-analysis of 14 eligible studies and 91,000 patients observed that patients with low serum vitamin D were 80% more likely to acquire COVID-19 infection as compared to those who have sufficient vitamin D levels (OR = 1.80; 95%CI: 1.72, 1.88) (Teshome et al., 2021). This is a remarkably similar finding to that described in a further systematic review and meta-analysis which evaluated data from 16 individual studies and 12,000 patients. Notwithstanding the methodological heterogeneity of the studies, in those that were adjusted (OR: 1.77; 95% CI: 1.24, 2.53) and non-adjusted (OR: 1.75; 95% CI: 1.44, 2.13) for confounders there was a higher risk of SARS-CoV-2 infection in the vitamin D deficiency groups (Kazemi et al., 2021). Thus a majority of studies support an association between low Vitamin D status and SARS-CoV-2 positivity ***.***

### What is the Association Between Vitamin D Status and COVID-19 Severity?

Many groups continue to observe that lower vitamin D status is associated with increasing severity of COVID-19 disease from asymptomatic to symptomatic, to respiratory failure, to a requirement for mechanical ventilation to death. In Germany, *Radujkovic et al.* studied the impact of vitamin D status on the severity of COVID-19 symptoms in 185 patients (half of whom were admitted to hospital). Most (64%) had a serum 25(OH) D less than 50 nmol/L and inpatients in particular had significantly lower median levels of vitamin D than outpatients (36.5 nmol/L vs. 46.5 nmol/L; *p* < *0.001*). Those with low vitamin D on admission had a higher risk of requiring invasive mechanical ventilation (HR = 6.12; *p < 0.001*) and a higher mortality rate (HR = 14.73; *p < 0.001*). In India, of 154 patients infected with SARS-CoV-2, 63 patients with severe symptoms had significantly lower serum vitamin D (mean = 36 nmol/L) at the time of admission compared to the remaining 91 asymptomatic infected patients (mean = 70 nmol/L) (*p = 0.0001*). Only two critical patients had normal levels of serum 25(OH)D3 (Jain et al., 2020). In an Austrian study of vitamin D levels in ICU patients only 4 of 26 patients who were admitted to ICU due to severe COVID-19 had adequate serum vitamin D levels on admission to hospital (Notz et al., 2021).

In a 2021 meta-analysis of data from fifteen studies, a higher composite disease severity was seen in patients with low vitamin D levels [adjusted (OR: 2.57; 95% CI: 1.65, 4.01; *I* ^2^ = 0.0%) and non-adjusted for confounders (OR: 10.61; 95% CI: 2.07, 54.23; *I* ^2^ = 90.8%]. Analysis of studies with crude OR’s (OR: 2.62; 95% CI: 1.13, 6.05; *I* ^2^: 47.9%) and adjusted studies that used the Cox survival method (HR: 2.35; 95% CI: 1.22, 4.52; *I* ^2^: 84%) indicated a significant association of vitamin D deficiency with mortality, while in adjusted studies that used logistic regression no relation was observed (OR: 1.05; 95% CI: 0.63, 1.75; *I* ^2^: 76.6%) (Kazemi et al., 2021). The observation is not explained by race. In one study of white patients, the majority (59%) of patients infected with SARS-CoV-2 who required hospitalisation for severe COVID-19 pneumonia had low serum 25(OH)D (<50 nmol/L) on admission and this was associated with subsequent COVID-19 mortality (De Smet et al., 2021).

### Is an Association Between Vitamin D Status and COVID-19 Biologically Plausible?

**FIGURE 2:**
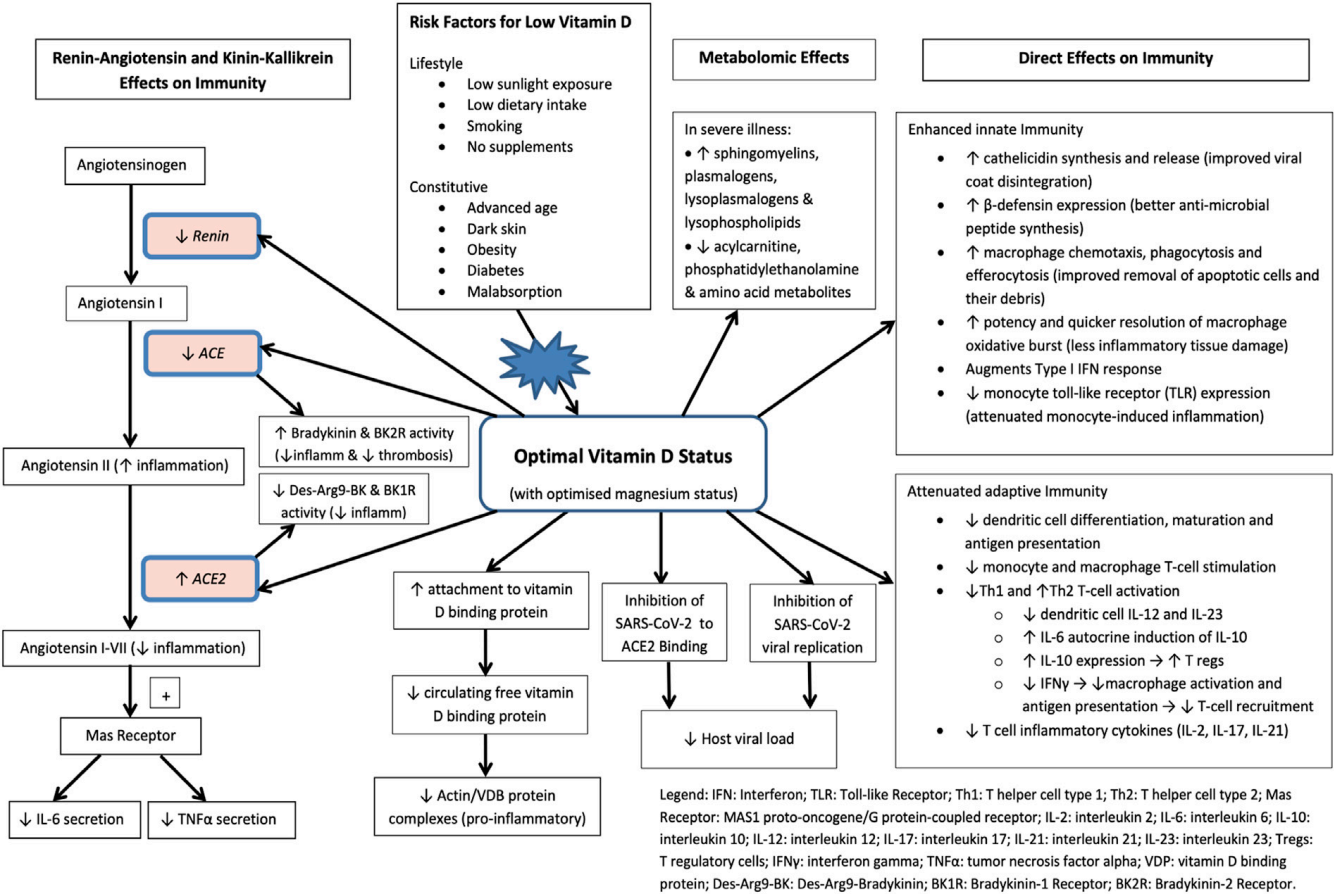
Molecular pathways in the pathology of Covid-19 thought to be affected by vitamin D (adapted from McCartney et al., 2020).

A biologic rationale exists for vitamin D in immune regulation (Figure 2) (McCartney et al., 2020). Apart from vitamin D’s proposed direct inhibitory effects on SARS-CoV-2 viral replication *in vitro* (Mok et al., 2020), *in silico* modelling has also suggested direct stearic hindrance of SARS-CoV-2 viral docking to the ACE2 receptor by vitamin D3 and its hydroxylated metabolites (Song et al., 2021). Further *in silico* modelling, subsequently confirmed by enzymatic inhibition assay, has also demonstrated that vitamin D and its lumisterol metabolites directly suppress the activity of SARS-CoV-2 main protease (Mpro) (by 10–19%) and SARS-CoV-2 RNA-dependent RNA polymerase (RdRP) (by 50–60%) (Qayyum et al., 2021). These findings suggest that 25(OH)L3, 24(OH)L3, 20(OH)7DHC and other hydroxylated vitamin D metabolites directly impede the activity of two SARS-CoV-2 transcription machinery enzymes which have critical roles in facilitating viral replication and the establishment of SARS-CoV-2 infection. Immunologically, 1.25(OH)_2_D3 can induce normal circulating mononuclear cells to differentiate towards monocyte-macrophages (Bartels et al., 2014). Vitamin D also influences the innate antibacterial element, β-defensin 2, which contributes to host defence by stimulating the expression of antiviral cytokines and chemokines involved in the recruitment of monocytes/macrophages, natural killer cells, neutrophils and T cells (Beard et al., 2011; Kim et al., 2018; Bishop et al., 2020). The possibility that vitamin D deficiency increases disease severity amongst some patients infected with SARS- CoV-2 is also biologically plausible, given the known association between vitamin D deficiency and elevated serum interleukin 6 (IL-6) (Miroliaee et al., 2018). A low vitamin D state is closely associated with elevated IL-6 in a variety of clinical settings. For example, significantly higher levels of serum IL-6 associated with vitamin D deficiency have been described in a variety of patients with various disorders ranging from diabetes, to osteoarthritis, to HIV/AIDS, to pancreatic ductal adenocarcinoma. While an increased serum IL-6 has long been associated with severe sepsis in humans, it is now linked to severe COVID-19 (Jain et al., 2020). Severe COVID-19 is particularly associated with pronounced elevations of IL-6, and IL-6 blockade has proven efficacy in severe disease. A number of groups have described associations between elevated IL-6 and worse outcomes after COVID-19. Moreover, the IL-6 blockers tocilizumab and sarilumab have shown some efficacy in COVID-19, supporting a specific role for IL-6 in the progression of disease. More recently, the role of vitamin D in facilitating transition from the pro-inflammatory Th1 phenotype which characterises severe COVID-19 disease, to the tolerogenic Th2 phenotype *via* IL-10 induction and its suppressive effects on IFNγ in the complement-rich micro-environment of SARS-CoV-2 infected lung epithelium has been elucidated, further supporting vitamin D’s causal role in ameliorating the hyper-inflammation observed in severe COVID-19 disease (Chauss et al., 2022). Together, these observations support the hypothesis that a low vitamin D status plays an important role in the progression of disease, in part through elevations in serum IL-6 and prolonged IFNγ elevation. Severe Covid is associated with angiotensin-converting enzyme 2 (ACE2) suppression and increased coagulability (Ragab et al., 2016; Rhodes et al., 2021); immunological and biochemical signatures which can be attenuated by vitamin D. Furthermore, there is a profound dysregulation of both the innate and adaptive immune systems with advancing age, with some functions down-regulated, and others up-regulated or even enhanced which contribute to the severity of responses to SARS-CoV-2 in older adults. (Manion et al., 2017; Kadijani et al., 2021; Rasmussen et al., 2021; Rezaei et al., 2021; Xiaohua et al., 2021).

### Vitamin D: Applying the Bradford Hill Criteria for Causation

The case for a low vitamin D state being relevant in SARS-CoV2 infection is supported by Bradford Hill Criteria for causation: first, a strong association exists between low vitamin D status and both the risk of SARS-CoV-2 infection (Kaufman et al., 2020; Merzon et al., 2020) and the severity of COVID-19 disease (Jain et al., 2020); second, the association is consistently found by a variety of research teams in many countries and in a variety of settings (Brown, 2020; McCartney and Byrne, 2020; McCartney et al., 2020 ); third, the association is specific and persists even in the absence of other potential influencing factors (Kaufman et al., 2020; Radujkovic et al., 2020); fourth, the presence of a low vitamin D state precedes the onset of the illness (rather than the reverse) (Annweiler et al., 2020; Meltzer et al., 2020b); fifth, a “biological gradient” appears to exist whereby more severe disease and mortality are associated with progressively lower vitamin D status (Baktash et al., 2020; Faul et al., 2020; Radujkovic et al., 2020); sixth, the known effects of vitamin D on immune cells provide a biologic plausibility that adequate levels of vitamin D may protect against severe disease (Greiller and Martineau, 2015; Bilezikian et al., 2020); seventh, the association agrees with studies which already show a relationship between vitamin D supplementation and reduced risk of other respiratory infections (Martineau et al., 2017; Jolliffe et al., 2021); and finally some emerging data show favourable outcomes in vitamin D therapy trials in COVID-19 patients (Entrenas Castillo et al., 2020; Ling et al., 2020). Although such intervention studies are notoriously difficult to design and interpret, *post hoc* analyses essentially discount the possibility that the more favourable outcomes of vitamin D-supplemented patients in the Cordoba study were attributable to chance or methodological issues such as imperfect blinding or uneven distribution of pre-existing comorbidities (Jungreis and Kellis, 2020).

### Might Vitamin D Prove a Therapy for Established COVID-19?

While a low vitamin D state is associated with the development of severe COVID-19 disease, a separate concept is that vitamin D might have a therapeutic effect in patients with symptomatic disease. The design of vitamin D randomised controlled trials (RCTs) is notoriously problematic and these trials commonly fail for a number of reasons (Boucher, 2020). First, vitamin D is a nutrient, rather than a drug. Withholding vitamin D in a “control” group could be harmful to study participants with a low vitamin D state, especially when the current data link a low vitamin D state with poor outcomes in COVID-19 and in other disease states; therefore, there are ethical impediments to randomised, placebo-controlled studies in this area. Second, in most RCTs, no dose is given to the “untreated” arm. In the case of vitamin D, unintentional dosing may occur through diet, the taking of supplements, or even exposure to sunlight (Wacker and Holick, 2013). In the well-designed VIDA study of vitamin D in asthma, for example, 13% in the vitamin D3 treated group and 15% of subjects in the “placebo” group reported taking supplements containing vitamin D at the end of the trial, making between-group differences more difficult to interpret (Castro et al., 2014). Indeed, 9% of subjects in the “placebo” group had serum 25(OH) D levels greater than 75 nmol/L at 12 weeks (Castro et al., 2014). Third, in most RCTs, identical doses are given to everyone in the treatment arm, but the doses may be too small to normalise serum 25(OH)D in many subjects with a low vitamin D state, and even large doses would be unlikely to induce significant changes in outcomes in “replete” subjects. Bolus doses also stimulate the fibroblast growth factor 23 (FGF 23)/24-hydroxylase counter-regulatory enzymatic pathways, meaning daily modest dosing is probably more effective than infrequent high dose bolus administration. The effects of vitamin D therapy may therefore be obscure, unless the results can be selected out for those who were deficient at baseline and ideally, successfully made replete during the RCT by daily or weekly dosing rather than more infrequent bolus doses (Boucher, 2020). Most RCTs, however, are designed and powered to observe effects (or lack of effect) across an entire study population. The need to allow for this confounder is well-illustrated by a study of individual participant data retrieved from 25 previous RCTs (approximately 11,000 subjects) for reduction of upper respiratory tract infection rates by treatment with various doses of vitamin D. The overall hazard ratio (HR) was 0.88 (95% CI; 0.81–0.96) but for subjects with a baseline 25(OH)D less than 25 nmol/L it was 0.30 (95% CI; 0.17–0.53), confirming the biologic principle that extra doses of vitamin D are not beneficial in subjects who are replete but are beneficial to those who have a low vitamin D state. Fourth, the threshold between “low vitamin D” and “replete” can vary according to the disease studied. The serum 25(OH)D threshold above which bone health, for example, is generally accepted as being protected is at least 50 nmol/L (Ross et al., 2011). In contrast, abnormal insulin resistance in non-diabetic subjects with a low vitamin D state is not reduced unless 25(OH)D values reach 80 nmol/L (Von Hurst et al., 2010).

The observed associations between low vitamin D and SARS-CoV-2 are consistent with findings in other respiratory illnesses. Data from 21,000 subjects across eight observational studies indicate that those with a serum vitamin D level <50 nmol/L have a 64% increased risk of community-acquired pneumonia (Zhou et al., 2019). As alluded to previously, recent meta-analyses indicate that vitamin D supplementation reduces the risk of acute respiratory infections generally, especially in people with the lowest serum 25(OH)D concentrations (Martineau et al., 2017; Jolliffe et al., 2021). These findings have recently been augmented by further meta-analysis of data indicating 3 to 4-fold reductions in risk of mechanical ventilation, ICU admission and mortality amongst hospitalised COVID-19 patients receiving vitamin D supplementation (Hariyanto et al., 2021).

### What is the Prevalence of Low Vitamin D Status in the Irish Population?

**FIGURE 3:**
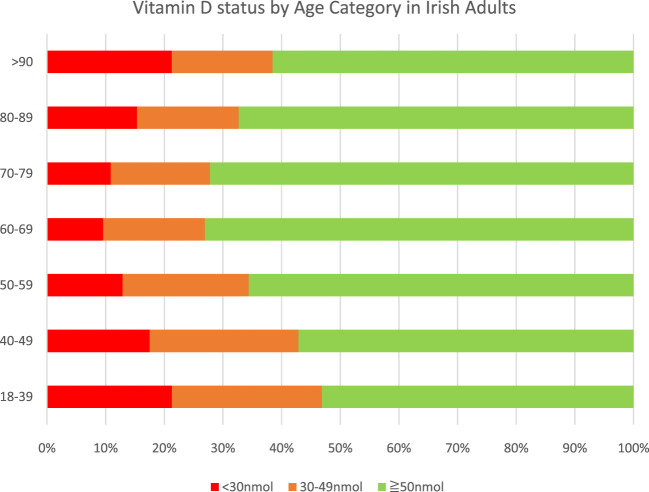
Prevalence of vitamin D deficiency and vitamin D levels <50 nmol/L in Irish adults (n = 36,466) (Adapted from Scully et al., 2020).

**FIGURE 4:**
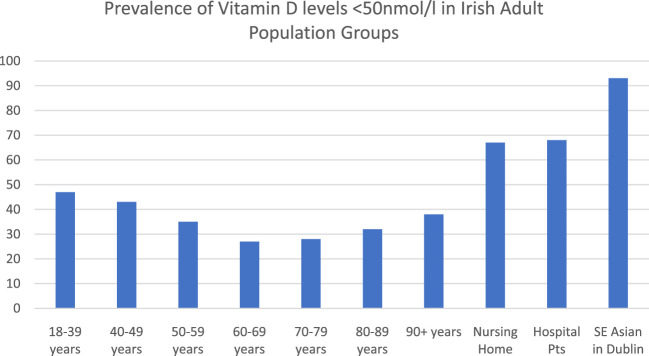
Prevalence of Vitamin D levels <50 nmol/L in Irish adult population groups. Adapted from Scully et al., 2020 (https://doi.org/10.3390/nu12092663); Griffin et al., 2020 (https://doi.org/10.1093/gerona/glaa010), Laird et al., 2020b (DOI: 10.3390/nu12123674).

Ireland is an island (situated between 51 and 56° North) with low levels of sunlight in wintertime, resulting in a high prevalence of low vitamin D status despite the presence of some voluntary food fortification. Serum 25(OH) D levels less than 50 nmol/L are seen in up to 50% of the Irish adult population in winter and 25% of the population during the summer (Scully et al., 2020). The overall prevalence of vitamin D levels below 50 nmol/L in Irish adults is presented in Figure 3 and Figure 4. The Irish Longitudinal Study on Ageing (TILDA) study, a representative population study of over 5,000 subjects aged over 50 years (mean age 64 years, range 50–96), reported that 45% of adults had serum 25(OH)D levels less than 50 nmol/L (Laird et al., 2018; Laird et al., 2020a). There was a notable seasonal and age gradient in low vitamin D status.

In a larger convenience study of 24,302 rural and urban patients from community (52 ± 16.4 years), outpatient (54.4 ± 17.9 years), inpatient (70.2 ± 17.5 years) and nursing home (81.5 ± 11.7 years) settings in the West of Ireland, over 50% of adults had serum 25(OH) D levels less than 50 nmol/L, rising to 67% of nursing home residents (Griffin et al., 2020). Vitamin D deficiency was more common in winter (December to February) and spring (March to May), in males, and in those aged ≥80 years.

In another large inner city convenience study of GP referrals, of whom 17,525 were aged over 50 years, low vitamin D status (<50 nmol/L) was evident in 40% (Scully et al., 2020). (Figure 3). In a study of 186 patients from Dublin’s Asian and Indian community (median age 32 years) only 6.7% had serum 25(OH) D levels greater than 50 nmol/L, with no significant increase during the summer months. (Laird et al., 2020b; Scully et al., 2020). None of the vitamin D levels cited in these papers are adjusted for supplement use. In the TILDA sample, 14% of women and 9% of men were taking supplements.

### Vitamin D Supplements and Food Fortification

Dietary supplements containing either vitamin D_2 (ergocalciferol)_ or D_3 (cholecalciferol)_ are widely available with and without prescription in Ireland. Vitamin D2 is manufactured using UV irradiation of ergosterol in yeast, and vitamin D3 is produced with irradiation of 7-dehydrocholesterol from lanolin and the chemical conversion of cholesterol (Holick et al., 2011). Vitamin D3 for vegans and vegetarians can now also be made from lichen and algae. Both forms are hydroxylated in the liver to form 25-hydroxyvitamin D2 (25(OH)D_2)_ and D3 (25(OH)D_3)_ respectively. Vitamin D3 increases serum 25(OH)D levels to a greater extent and maintains high levels longer than vitamin D2 (Tripkovic et al., 2012; Tripkovic et al., 2017).

In Ireland physiological vitamin D requirements for optimal immune function are considerably higher than those that can be achieved from food alone. Currently, most countries in Europe recommend daily vitamin D intakes of 600–800 international units (IU) for older age groups (European Food Safety Authority Panel on Dietetic Products, 2016). In the USA, the IOM, now the National Academy of Medicine (NAM) and the Endocrine Society recommend daily vitamin D intakes of 800 IU and between 1500 and 2000 IU respectively (Lips et al., 2019). The Food Safety Authority of Ireland (FSAI) recommends that for older adults living independently and who get exposure to sunlight during summer, a daily supplement of 600 IU of vitamin D should be taken during the extended winter months for bone health (i.e. the prevention of metabolic bone disease and osteomalacia) (FSAI, 2020).

However, in Ireland, the oral daily dose of vitamin D required to maintain serum levels above 50 nmol/L in 97.5% of healthy Irish adults throughout the year has been calculated to be 1120 IU (Cashman et al., 2008) with an oral daily dose of 988 IU required for adults aged 64 years and over (Cashman et al., 2009). The 2011 National Adult Nutrition Survey (NANS) reported that 27% of adults older than 65 years (males: 21%; females: 32%) took a nutritional supplement containing vitamin D (Kehoe et al., 2017).

Natural dietary sources of vitamin D include oily fish such as salmon, tuna, mackerel, sardines, herring, cod liver oil, shiitake mushrooms and meat such as liver and beef. Wild fish have higher concentrations of vitamin D than farmed. Food sources fortified with vitamin D include fortified milk, yogurts, breakfast cereals and infant formula (Table 1.). The 2011 NANS reported mean daily intakes of vitamin D from all sources (diet and supplements) of just 5.2 μg (208 IU) for men and 8.5 μg (340 IU) for women. Within the TILDA cohort (mean age 64 years) the proportion of the population who take supplements have significantly higher 25(OH)D concentrations and a lower prevalence of low vitamin D status throughout both summer and winter than their non-supplementing peers. Vitamin D supplement use is higher among women than men (12.6 vs 4.3%) and among the non-obese versus obese (10 vs 5.9%). A recent survey of supplement use during the pandemic (July–November 2020) confirmed that while vitamin D supplement use in TILDA participants had increased from 9 to 11.1% for men and 14–17.3% for women, the overall prevalence of supplementation remains low.

**TABLE 1:** Natural dietary and fortified food sources of Vitamin D.

Natural foods			
Food	Quantity	Vitamin D	
		μg	IU
Salmon	100 g	2.9–18.5*	116–740
Wild		9.4–18.5	376–740
Farmed		2.9–9.5	116–380
Trout	100 g	10	400
Mackerel	100 g	8.6	344
Herring	100 g	5.4	216
Sardines	100 g	5	200
Tuna	100 g	3	120
Beef liver (cooked)	100 g	3.7	148
Cod liver oil	15 ml	34	1,360
Eggs	2 eggs	4	160
Mushroom (Shiitake)	100 g	3.9	156
Milk	200 ml	2–4	80–160
Orange Juice	150 ml	2.5	100
Infant formula	100 ml	1.7	68
Edible oils/spreads	100 g	5.5	220
Cheese	One cheese string	1.3	52
Fortified Cereal	30–40 g	1.5–2.9	60–116
Wholegrain Bread	2 slices	0.8	32
Yogurt	125 g	0.8–5.0	32–200

Μg, Micrograms; IU, international units.

a most of the salmon consumed in Ireland is farmed salmon. Approximation only: refer to nutrition labelling as the amounts of vitamin D added to fortified foods changes regularly.

### Benefits From Food Fortification and Supplementation in Finland and Relevance to COVID-19

In selected European countries, low serum 25(OH)D concentrations appear to be associated with increased mortality from COVID-19 (Laird et al., 2020a). Moreover, countries with a formal vitamin D fortification policy appear to have the lowest rates of infection whilst countries with no policy and the highest deficiency rates appear to have higher infection and COVID-19 mortality rates. A significant negative correlation exists between historical mean vitamin D concentrations in European countries and SARS-CoV-2 positivity and COVID-19 mortality per million of population (Ilie et al., 2020). The introduction of mandatory fortification of food products with vitamin D (as practiced in some Nordic countries) and the promotion of increased dietary intake of vitamin D rich foods is both safe and has the potential to significantly reduce the prevalence of low vitamin D status in a population (Jääskeläinen et al., 2017). In 2003, Finland implemented a Public Health Initiative to increase vitamin D supplementation and increase vitamin D fortification of fluid milk products and fat spreads with the aim of improving population vitamin D status. This strategy resulted in an increase in the mean adult serum 25(OH)D levels from 48 nmol/L (95% CI: 47, 48 nmol/L) to 65 nmol/L (95% CI: 65, 66 nmol/L) (*p < 0.001*) and achieved an increase in the prevalence of vitamin D supplement users from 11 to 41% (*p < 0.001*). In 2011, a mere 8 years post the introduction of this strategy, 91% of non-vitamin D supplement users who consumed fluid milk products, fat spreads, and fish based on Finnish nutrition recommendations reached 25(OH)D concentrations greater than 50 nmol/L. Based on the current voluntary fortification practices in Ireland, one would need to consume either four bowls of fortified cereal, or 6 eggs, or 4 tins of sardines each day to maintain an oral intake of 10 μg (400 IU). Since sunlight and food sources of vitamin D do not provide adequate vitamin D for older adults in Ireland, and current voluntary fortification practices, while helpful, are ineffective on their own in terms of achieving adequate intakes in the population, it is important that the population take vitamin D supplements of sufficient dose to help avoid a low vitamin D state.

### Summary and Recommendations

A low vitamin D state is often used to describe patients at risk of bone disease (25(OH)D < 50 nmol/L) rather than infection. It remains unknown what constitutes “low” in terms of a vitamin D state that protects against infection. Not only is it likely that the threshold for bone health is different to that for immune health, it seems likely that much higher serum vitamin D levels (possibly greater than 100 nmol/L are required to protect against infection with SARS-CoV2 (Spedding et al., 2013). A low vitamin D status, commonly seen in older adults, the obese, those with dark skin pigmentation, those with diabetes mellitus, residents of assisted care facilities, those living at higher latitudes (Keane et al., 1998; Spiro and Buttriss, 2014; Rhodes et al., 2021) and other groups is linked to increased SARS-CoV-2 positivity and to the development of severe COVID-19 disease in a variety of countries including Ireland (Ali, 2020; Faul et al., 2020; Grant et al., 2020; McCartney and Byrne, 2020; McCartney et al., 2020; Breslin et al., 2021; Connolly et al., 2021). Vitamin D supports immune mechanisms that protect against disease caused by SARS-CoV-2: chiefly by increasing secretion of anti-viral defensins (Beard et al., 2011; Kim et al., 2018), by regulating inflammatory responses through upregulated production of the anti-inflammatory cytokine IL-10 (Ramos-Martínez et al., 2018; Di Liberto et al., 2019 ) and by restoring lung ACE-2 production (Grant et al., 2020; Malek Mahdavi, 2020; Xiao et al., 2021). When the immune system is impaired in the setting of a low vitamin D state there is an increased risk of SARS-CoV-2 infection and of severe COVID-19 disease after infection, as now demonstrated in a large body of epidemiologic and clinical research.

A window exists in which a large number of individuals would benefit from vitamin D supplementation because their low vitamin D state places their immune system in a vulnerable position. This is especially important during the current pandemic. Vitamin D status is important, not only because of the wealth of epidemiologic and clinical data that now link low serum vitamin D levels to an increased risk and severity of SARS-CoV-2 infection, but also because an opportunity exists to treat a low vitamin D status which is highly prevalent in the Irish population. Public health messaging to the general population should be deployed to increase vitamin D supplementation rates and improve the vitamin D status of the population. A replete vitamin D state will support the immune response to this potentially deadly infection for which there is no effective treatment.

The recommended daily vitamin D intake (primarily for bone health) ranges from 400 to 600 IU, European Food Safety Authority (European Food Safety Authority, 2012) to 400–800 IU (Institute of Medicine, 2011), with even lower daily doses of 400 IU recommended in the United Kingdom by the Scientific Advisory Committee on Nutrition (SACN, 2016). Given average dose response effects (∼0.7 nmol/L increment in serum 25(OH)D per additional 40 IU of oral intake) (SACN, 2016) and prevailing population serum levels, it is unlikely that currently recommended intakes will achieve the serum vitamin D levels required to fully optimise immune function, including immune response to the SARS-CoV-2 virus. In this context, and in the absence of specific clinical contraindications (e.g. lymphoma, sarcoidosis, *tuberculosis* or other granulomatous disorders), daily supplementation doses of 800–1,000 IU/d and higher (for example 1,500–2000 IU/d as advocated by the Endocrine Society would appear reasonable for adults to reach immunologically-relevant levels of repletion in a timely manner. These doses may be particularly important for vulnerable population groups with limited cutaneous synthesis (e.g., older adults and those with darker skin pigmentation) or those whose response to oral dosing is attenuated (e.g., overweight or obese individuals). Furthermore, these doses are well within the consensus safe daily intake (tolerable upper limit) of 4000 IU/d defined by EFSA (2016), IOM/NAM (IOM, 2011) and Scientific Advisory Committee on Nutrition (2016).

We recommend that all adults ensure an intake of 800 to 1,000 units (20–25 μg) of vitamin D3 or more per day. People who are housebound or work indoors or have obesity or dark skin pigmentation may need a higher dose of vitamin D3 supplementation to replete their low vitamin D state (e.g., equivalent to the 1,500–2000 IU recommended by the Endocrine Society), in order to ensure that optimal 25(OH)D blood levels for immune function are reached. The use of serum 25(OH)D levels is a reasonable guide on vitamin D status. We strongly recommend that the Irish Department of Health and Health Service Executive adopt a policy of recommending increased vitamin D supplementation for all age groups not only during the COVID-19 pandemic but also on an ongoing basis (FSAI, 2020, Laird and Kenny, 2020).

## Key Points

i) Recommended Vitamin D intake 800–1000 IU (20–25 μg) per day, with higher monitored doses e.g., 1,500–2000 IU (37.5–50 μg) per day for vulnerable groups who have confirmed or likely low vitamin D status (e.g., those with obesity, darker skin, or in nursing homes or residential care).
ii) 50% of Irish adults in winter and 25% in summer have levels <50 nmol/L.
iii) Further risk factors for deficiency include not taking supplements, smoking, physical inactivity, living alone, low socioeconomic status and diabetes mellitus.
iv) Deficiency of Vitamin D has an association with severe COVID-19 - both SARS-CoV-2 infection and severity of Covid-19 disease are higher with insufficient blood levels.

## Data Availability

The data analyzed in this study is subject to the following licenses/restrictions: They are hospital laboratory data sets—Hospital Research Committee ethical permission has been acquired for their anonymous use. Requests to access these datasets should be directed to rkenny@tcd.ie.
